# Observational studies: why are they so important? Estudos observacionais: por que são tão importantes?

**DOI:** 10.1590/1516-3180.2014.1321784

**Published:** 2014-02-01

**Authors:** Alessandro Wasum Mariani, Paulo Manuel Pêgo-Fernandes

**Affiliations:** I MD. Thoracic Surgeon, Instituto do Coração (InCor), Hospital das Clínicas (HC), Faculdade de Medicina da Universidade de São Paulo (FMUSP), São Paulo, Brazil; II MD, PhD. Associate Professor, Discipline of Thoracic Surgery, Instituto do Coração (InCor), Hospital das Clínicas (HC), Faculdade de Medicina da Universidade de São Paulo (FMUSP), São Paulo, Brazil

Randomized clinical trials (RCTs) are known to be the gold standard for medical research, and this study design is preferred for investigating the efficacy of new interventions. Many authors and editors believe that the RCT design always surpasses other research designs. However, this is not an unquestionable truth.

The main advantage of RCTs is that they provide better control over possible bias through randomization and blinding. In other words, the great strength of an RCT is its high internal validity. On the other hand, rigid design control could reduce the ability to generalize the results.^1^ Another issue relating to RCTs is the fact that recruitment, randomization and blinding are not always possible because of technical issues (e.g. surgical procedures) or ethical issues (e.g. in a hypothetical trial to prove the association between smoking and lung cancer, it would be unethical to randomly assign a group to smoke).

But what should be done when RCTs are unfeasible or unethical? The answer could be a well-designed observational study. This term describes a range of study designs that includes cross-sectional, case-control, prospective and retrospective cohort studies. The main characteristic of observational studies is that any intervention is not determined by the protocol, but by clinical practice.^2^

Cross-sectional studies are used to estimate prevalence. They are relatively quick and cheap, and can be used to study multiple outcomes. However, they do not differentiate between cause and effect or within the sequence of events. They are useful for identifying associations that can then be more rigorously studied using a cohort study or randomized controlled study. The most important problem with this type of study is in differentiating between cause and effect from a simple association.^3^

Case-control studies are considered to be simple to organize and useful for hypothesis generation. They retrospectively compare two groups in order to identify predictors of an outcome. They allow calculation of odds ratios. The disadvantages are that they can only evaluate one outcome and that the presence of bias is usually high and difficult to assess. The major difficulty could lie in determining an adequate control group.^3^

Cohort studies are excellent for estimating the incidence and natural history of a condition. They may be prospective or retrospective and sometimes two cohorts are compared. They analyze predictors (risk factors) that enable relative risk calculation. Since they measure events in temporal sequence, they can distinguish causes from effects. Retrospective cohorts are considered to be cheaper and quicker, but may have fragile results, particularly if the database is inadequate. Prospective cohorts are more accurate and, with a good protocol (with adequate sample size and follow-up) can have results that are as reliable as those of RCTs.^3^

The main problem in observational studies is the presence of confounders and selection bias (which are prevented in RCTs through randomization and blinding). A confounder can be defined as any factor that is related not only to the intervention (e.g. treatment) but also to the outcome and could affect both.^4^ One good example is age: in a study on the relationship between smoking (exposure) and lung cancer (outcome), age could be implicated as a factor that would increase the incidence of the outcome. Thus, if one of the groups (smokers or non-smokers) has an older population, the increase in lung cancer could be influenced by age (as a confounder), and not by the exposure studied. 

However, advanced statistical tools may enable good and reliable control over many confounders. Some tools like propensity scores and sensitivity analysis, when correctly performed, could drastically reduce the bias caused by the lack of randomization.^5^

Some authors have studied the results from RCTs, compared with similar observational studies. Concato et al. published an evaluation of meta-analyses that compared outcomes between RCTs and observational studies and reached the following conclusion: "The results from well-designed observational studies (with either a cohort or a case-control design) do not systematically overestimate the magnitude of the effects of treatment, as compared with those in randomized, controlled trials on the same topic".^6^ In other words, if the observational study has good methodological quality, the results are quite similar.

Many investigators have pointed out that the main strength of observational studies is their greater proximity to "real life situations", since RCTs have stricter inclusion criteria and rigid protocols that may not reflect clinical practice. By definition, observational studies have greater heterogeneity of medical interventions and patient populations that are closer to clinical practice.^2^Other advantages of observational studies are that they are usually cheaper than RCTs and can be used to investigate rare outcomes and to detect unusual side effects, and that some designs are easily and quickly performed.

Observational studies also are important for creating new hypotheses, proving the external validity of RCTs already performed, establishing the sample size for an RCT and evaluating which patient subsets really benefit from each alternative intervention of effective alternative therapies.^1^ In this way, it can be said that observational studies can be complementary to RCTs.

Although the evidence level of observational studies appears to be lower than that of RCTs, it is clear that this kind of investigation is crucial for elucidating many scientific questions. Not only authors but also editors around the world are giving more attention to these studies. The take-home message is that the study question and the quality of the methodology applied to answer it are much more important than the study design. In this context, observational studies may be the best way to answer the many medical questions in situations in which the classical RCT approach does not apply. 
